# Individual differences in cognitive processing for roughness rating of fine and coarse textures

**DOI:** 10.1371/journal.pone.0211407

**Published:** 2019-01-30

**Authors:** Makiko Natsume, Yoshihiro Tanaka, Astrid M. L. Kappers

**Affiliations:** 1 Nagoya Institute of Technology, Department of Electrical and Mechanical Engineering, Nagoya, Japan; 2 JST, PRESTO, Kawaguchi, Japan; 3 VU Amsterdam, Department of Human Movement Science, Amsterdam, The Netherlands; University of Ottawa, CANADA

## Abstract

Previous studies have demonstrated that skin vibration is an important factor affecting the roughness perception of fine textures. For coarse textures, the determining physical factor is much less clear and there are indications that this might be participant-dependent. In this paper, we focused on roughness perception of both coarse and fine textures of different materials (glass particle surfaces and sandpapers). We investigated the relationship between subjective roughness ratings and three physical parameters (skin vibration, friction coefficient, and particle size) within a group of 30 participants. Results of the glass particle surfaces showed both spatial information (particle size) and temporal information (skin vibration) had a high correlation with subjective roughness ratings. The former correlation was slightly but significantly higher than the latter. The results also indicated different weights of temporal information and spatial information for roughness ratings among participants. Roughness ratings of a different material (sandpaper versus glass particles) could be either larger, similar or smaller, indicating differences among individuals. The best way to describe our results is that in their perceptual evaluation of roughness, different individuals weight temporal information, spatial information, and other mechanical properties differently.

## Introduction

When we evaluate the characteristics of an object, we attempt to obtain some tactile information by touching the object. The tactile information is based on deformations, vibrations, and temperature changes on the skin, which are elicited by the mechanical interaction between the finger and the object. As mechanical interaction with the skin depends not only on the material properties of the object but also on the individual characteristics of the skin and the contact conditions, there will be individual differences in the induced tactile sensations. Furthermore, cognitive aspects might cause differences in tactile sensation. Tanaka et al. [1] showed that the sensitivity to vibrotactile stimulation and the change of the sensitivity by increasing the skin vibration varied among participants. Hollins et al. [2] demonstrated that perceptual space comprises two dimensions: roughness/smooth and soft/hard for some participants, whereas for other participants, space comprises three dimensions: roughness/smooth, soft/hard, and sticky/slippery. Thus, tactile sensation is extremely complex and subjective. The aim of this study is to give a first insight into individual differences on tactile sensation (in particular surface roughness). Findings of the present paper could provide useful knowledge to achieve a better understanding of the relationship between physical parameters and the tactile sensation among individuals. They will not only be valuable from a fundamental point of view, but also be useful for the design of textures and the development of haptic sensors and displays.

Okamoto et al. [3] reported that the five most relevant dimensions of tactile sensation are hardness/softness, coldness/warmness, macro and fine roughness, and friction (which includes moistness/dryness and stickiness/slipperiness). It is particularly interesting that roughness perception is separated into two dimensions: macro and fine roughness, whereas we do not distinguish them in separate categories in daily life. Hollins et al. [4, 5, 6] determined that the border between macro and fine roughness lies around a texture element size of about 100–200 μm. Above this border, humans can discriminate the roughness of surfaces without moving their fingers (static perception); below this border, finger movements are necessary (dynamic perception). Regarding the perception of macro textures, Hollins and Risner [4] showed that the ability to discriminate coarse textures is equal in moving and stationary conditions. In this paper, we focus on roughness perception including both macro and fine textures.

Studies on roughness perception have been conducted with a focus on spatial information, friction, and skin vibration (as a parameter of temporal information), and fine and/or coarse textures were used in the studies. The relationship between spatial information and roughness perception was studied in classical experiments using coarse textures with a periodic surface. It was demonstrated that roughness estimation increases as the groove size widens [7, 8, 9, 10]. In industry, various roughness parameters like *Ra* (arithmetic mean roughness) and *Rq* (root-mean-square height) are used as measures for physical roughness. These parameters are based on the height profile of the surface and they represent certain surface characteristics. Bergmann Tiest and Kappers [11] examined the relationship between some spatial-based roughness parameters and subjective roughness for various textures including both fine and coarse textures. They concluded that the parameter that most strongly correlates with subjective roughness depends on both the participant and the modality (touch or vision). Regarding friction, Ekman et al. [12] reported that there is a relationship between the friction coefficient and subjective roughness. Smith et al. [13, 14] conducted an experiment on roughness estimation with coarse textures having a periodic surface. They demonstrated that frictional force correlates with subjective roughness. On the other hand, Taylor and Lederman [9] concluded that there is no relationship between the friction coefficient and subjective roughness for coarse periodic surfaces. As the different groups used different textures, more research is required to understand the inconsistent findings which have been obtained when studying the friction coefficient.

Recently, studies that focused on skin vibration as tactile information were conducted. Bensmaia and Hollins [15] recorded skin vibrations using a Hall effect transducer and showed that there is a correlation between skin vibration and the roughness of fine-textured surfaces including daily-life materials. They computed the vibration weighted with the sensitivity of Pacinian receptors and concluded that the textural information of fine surface textures is represented by Pacinian corpuscles. Delhaye et al. [16] reported that the intensity of the vibration measured at the wrist is related to the scanned texture of periodic surfaces and sandpapers. Tanaka et al. [17] developed a wearable skin vibration sensor and showed that the skin vibration increases with increasing grain size of sandpapers [18]. These studies show the possible contribution of skin vibration to the perception of fine textures and support the finding that movement is required for fine roughness discrimination. Other related psychophysical works [19, 20, 21] supported vibrational cues affecting fine roughness. Skin vibration on coarse textures has been also investigated. Gamzu and Ahissar [22] reported that temporal information is useful for discrimination of periodic coarse surfaces. Gescheider and Wright [23, 24] reported some Pacinian corpuscle mediation on the perception of coarse texture roughness. Manfredi et al. [25] reported that various textures, which include both fine and coarse textures, can be sorted by the skin vibration measured with a laser-Doppler vibrometer. The present authors [26] conducted an experiment using a wide variety of materials and showed that subjective roughness values are related to skin vibration. From a neuroscience point of view, Yoshioka et al. [27] demonstrated that spatial variation in the firing rates of slowly adapting type 1 afferents accounts for the perceived roughness of grating patterns (groove widths of 0.1–2.0 mm) and that Pacinian afferents make no contribution to encoding the roughness of these textures. Lieber et. al. [28] showed that a variation-based neural code can account for roughness perception. Weber et al. [29] proposed a combined model using spatial (slowly adapting type 1 afferents) and temporal (Pacinian and rapidly adapting afferents) information to account for the roughness perception of fine and coarse textures.

The mechanisms of roughness perception remain a controversial topic. Apparently, the availability of temporal information is not a prerequisite, which indicates that spatial information is the dominant factor in the discrimination of coarse textures. On the other hand, some studies indicate that temporal information can also contribute to roughness perception. In addition, other parameters like friction might influence roughness perception. Fine and coarse textures have different friction coefficients depending on the profile and material of the surface. Although we spontaneously evaluate various textures without categorizing them into coarse and fine textures, there are hardly any studies that investigate the influence of spatial, frictional, and temporal information on roughness ratings for both fine and coarse textures. We are especially interested in possible individual differences on the roughness ratings in daily lives, as it might be the case that participants focus on different aspects (temporal, spatial, friction, or others) of the stimulus. Therefore, in this study, glass stimuli of various particle sizes covering both coarse and fine textures are used. In addition, also sandpapers, which can be categorized as fine textures based on their profiles, are used. Properties like friction and edges are very different for sandpapers as compared to those of glass particle surfaces. To investigate the natural perception of roughness, this study investigates the relationship between roughness perception and various physical parameters by asking individuals to rate the roughness of various surfaces. We measure skin vibration, friction coefficient, and texture element size as temporal, frictional and spatial information, respectively.

## Methods

### Participants

Thirty healthy adults (18 males and 12 females, ages 18–24, listed as P1–P30) participated in the experiment. We assessed their dominant hand according to Coren’s test [30]. Twenty-eight of the participants were strongly right-handed, one was moderately left-handed and one was strongly left-handed. They used the index finger of their dominant hand during the experiment and were naive about the purpose of our experiment. All participants gave their written informed consent before participating in the experiment and they were paid for their time. The experiment was approved by the Ethics Committee of Nagoya Institute of Technology.

### Stimuli

**Fig 1:**
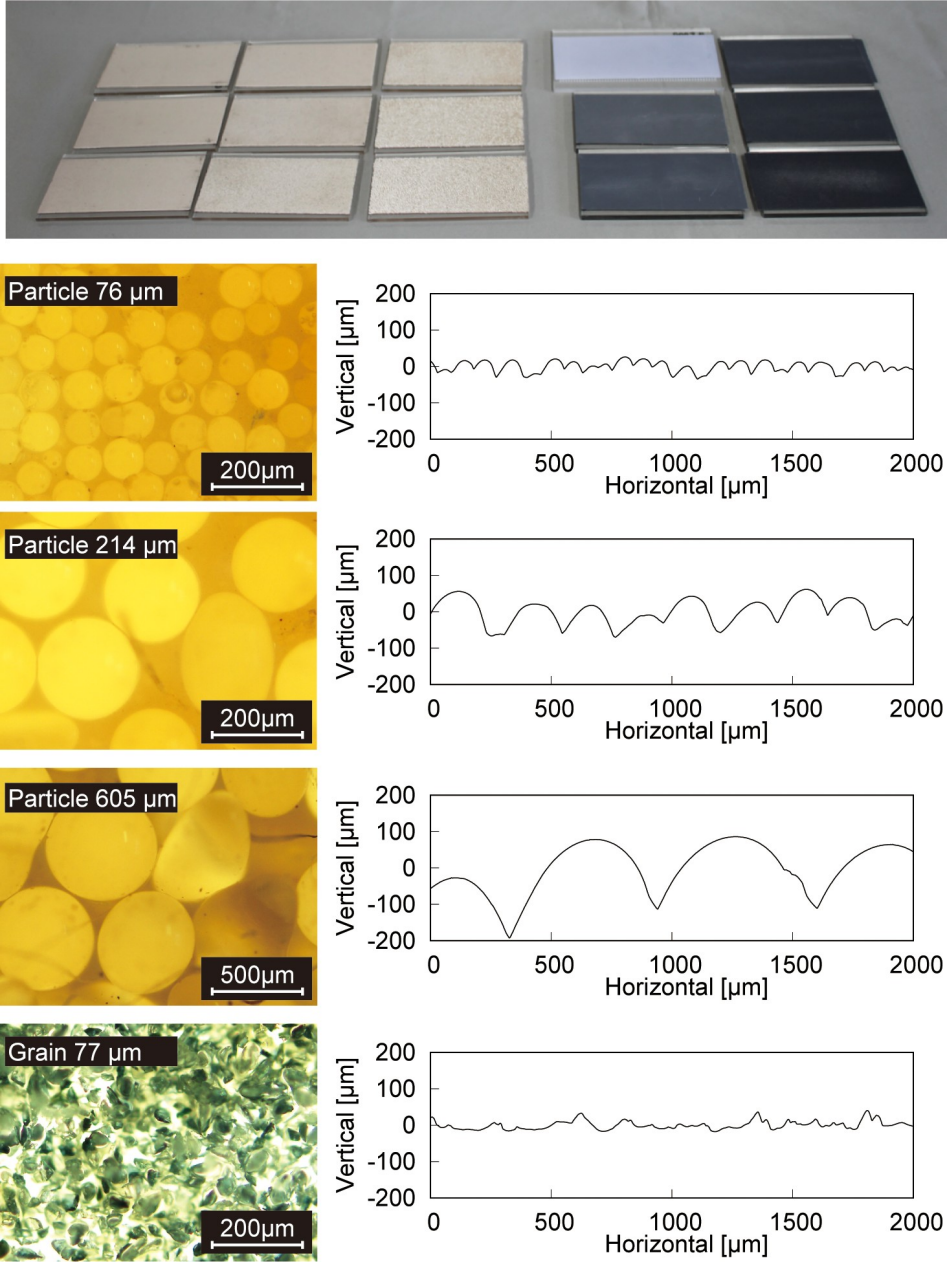
Stimulus set. Top: Photo overview of all the stimuli. Bottom: Examples of magnified photographs and surface profiles of three glass particle and one sandpaper stimuli.

We prepared 15 plates as shown in Fig 1; each plate was made with one of two types of surfaces. Nine of the surfaces were made from glass particles of different sizes, and the other six were made from sandpapers of different grain sizes. The glass particles were attached to a flat 10 x 6 cm acrylic plate with double-sided tape similar to how Tsuboi et al. [31] did in their research on micro-particulate plates. The particles did not have any sharp edges. In the present paper, textures with an average particle size smaller than 200 μm are considered as fine, and textures with particle sizes larger than 200 μm are considered as coarse. The sandpapers were employed as stimuli having different properties. They were also attached to acrylic plates and were made from silicon carbide that has sharp edges. All sandpapers were categorized as fine textures because of their grain size. Table 1 shows the average particle or grain size of each stimulus. For the glass particles, the numbers within parentheses indicate the range of the particle size of the stimulus as certified by the distributor (KENIS LIMITED). For the sandpapers, the numbers indicate the average grain sizes. Fig 1 shows magnified photographs and surface profiles of some of the stimuli. The profiles were measured with a roughness tester (Mitutoyo Corporation). Photographs and profiles of all stimuli are shown in S1 Fig.

**Table 1:** Average particle or grain size of the stimuli. The range of particle sizes is shown inside the parentheses.

**Glass particle**	**Average particle size [μm]**	**Sandpaper**	**Average grain size [μm]**
	50 (37–63)		1
	76 (63–88)		20
	115 (105–125)		58
	163 (149–177)		67
	214 (177–250)		77
	425 (350–500)		94
	605 (500–710)
	850 (710–990)
	1194 (991–1397)

### Experimental set-up

**Fig 2:**
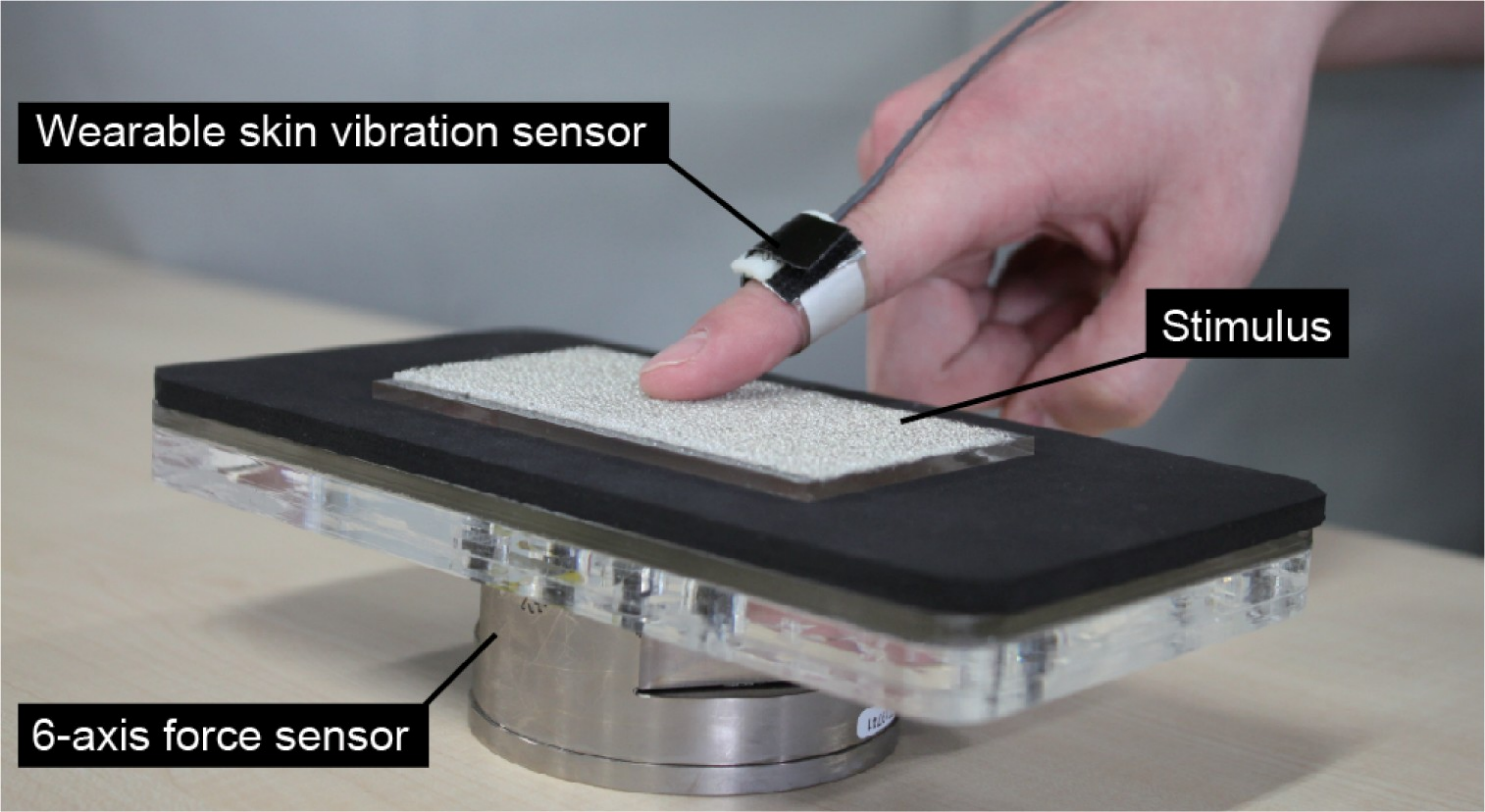
Experimental set-up.

Fig 2 shows the experimental set-up. For measuring skin-propagated vibrations, we used a wearable skin vibration sensor developed by Tanaka et al. [17]. The sensor allows fingertips to touch an object directly. The vibration elicited by rubbing an object is propagated on the skin, and the sensor measures the skin-propagated vibrations. Since a PVDF (polyvinylidene difluoride) film, which is very flexible and light, is applied to this sensor, the skin vibration can be measured without limiting the exploratory movement. The sensor responds to tension applied by skin-propagated vibration. Thus, the sensor output associates with acceleration. We already demonstrated that the sensor outputs correlate with grain size [18] and roughness perception [26].

In this paper, the sensor was placed on the middle phalanx of the index finger, and it was strapped at a similar tension for each participant [32]. For measuring the exerted force, the stimuli were placed on a 6-axis force sensor (see Fig 2).

### Procedure

The main experimental task for participants was to rate the subjective roughness with a method similar to that used in our previous study [26]. Participants were instructed to rub the presented stimulus three times from left to right (in the case that their dominant hand is the right hand) and to use more or less the same velocity and force every time for each stimulus. Glass particle surfaces of fine and coarse textures and sandpapers were pseudorandomly given to participants for the test of roughness ratings. As reference stimuli for the ratings, we presented two sandpapers whose grain sizes were the smallest and largest of the sandpaper plates, namely 1 and 94 μm, respectively. We told the participants that the smooth one had a roughness of 1 and the rough one had a roughness of 100. Participants rated the roughness of all stimuli by giving a value based on the reference stimuli. They could not use negative numbers, but they could choose a value higher than 100 if they felt a stimulus was rougher than the reference stimulus of roughness 100. During the experiment, participants were blindfolded so that they could not see the presented stimuli, and they wore headphones so that they could not hear the sound caused by touching the stimuli.

For each participant, the experiment consisted of five sessions; in each session, all 15 stimuli were tested and the first and second stimuli were always the smooth stimulus with roughness 1 and the rougher stimulus with roughness 100, respectively. The third through fifteenth stimuli were presented in random order. The sampling frequency of both sensors was 10 kHz. A low-pass filter with a cutoff frequency of 10 Hz and a high-pass filter with a cutoff frequency of 10 Hz were used for measuring forces and skin vibration, respectively. The first session was conducted for practice.

### Data processing

#### Sensor output

Sensor output signals during the stable rubbing period were extracted from the collected data. The stable period corresponded to the 0.3 s within one stroke from 0.15 s before to 0.15 s after the center point between the start and end points of rubbing; this was also defined as the stable rubbing period in our previous study [26]. We calculated the sum of the power spectral density on the data of sensor output as an evaluation parameter that indicates the intensity of the skin vibration. The intensity of the skin vibration *V* was calculated from the data for each stable rubbing period by

$$V=\log_{10}\frac{\int_{10}^{1000}PSD_{rub}\left(f\right)df}{\int_{10}^{1000}PSD_{noise}\left(f\right)df},$$ (1)

where *f* is frequency, *PSD*_*rub*_ is the power spectral density calculated from the data of the sensor output during stable rubbing, and *PSD*_*noise*_ is the power spectral density calculated from the data of the sensor output when there is no contact with an object. *PSD*_*noise*_ was used for excluding the noise of the sensor output. Previous psychophysical and neuroscience works [4, 15, 19, 20, 21, 28] found that temporal information is one of the important factors for the perception of fine textures and that the roughness information is represented by Pacinian afferents. Mountcastle et al. [33] found that Pacinian corpuscles can strongly react to stimuli of 60 Hz or above and Connor et al. [34] demonstrated that these receptors are unresponsive to spatial information. However, in the model by Weber et al. [29] both Pacinian afferents and rapidly adapting afferents were employed, and even slowly adapting type 1 afferents respond to temporal stimulation. Moreover, the authors’ previous work demonstrated that the intensity of the skin vibrations between 10 and 1000 Hz correlates well with roughness ratings [26]. Temporal cues for roughness perception are still under investigation. For these reasons, we choose as a possible interval the data obtained from 10 Hz to 1000 Hz to determine the temporal information of skin vibration. The average intensity of three strokes was calculated for each sample. For the exerted force, the average of all the extracted data during the stable periods was calculated for each stimulus. The contact force was computed from the vertical force, and the tangential force was calculated by

$$F=\sqrt{F_{x}{}^{2}+F_{y}{}^{2}},$$ (2)

where *F*_*x*_ and *F*_*y*_ are the horizontal forces on the contact surface. The friction coefficients of the samples were calculated by dividing the average contact force by the average tangential force during each stable stroke.

#### Curve fitting

To evaluate the relationship between subjective roughness ratings and skin vibration or particle size for each parameter, the following power function was fitted to the ratings of the fine and coarse glass particle surfaces:

$$Y=aX^{b},$$ (3)

where *Y* is the subjective value, *X* is the parameter considered, and *a* and *b* are the values obtained by the least squares fitting method. For this fit, a power function was chosen since Stevens [35] proposed that magnitude estimations increase in proportion to the stimulus intensity raised to a power.

**Fig 3:**
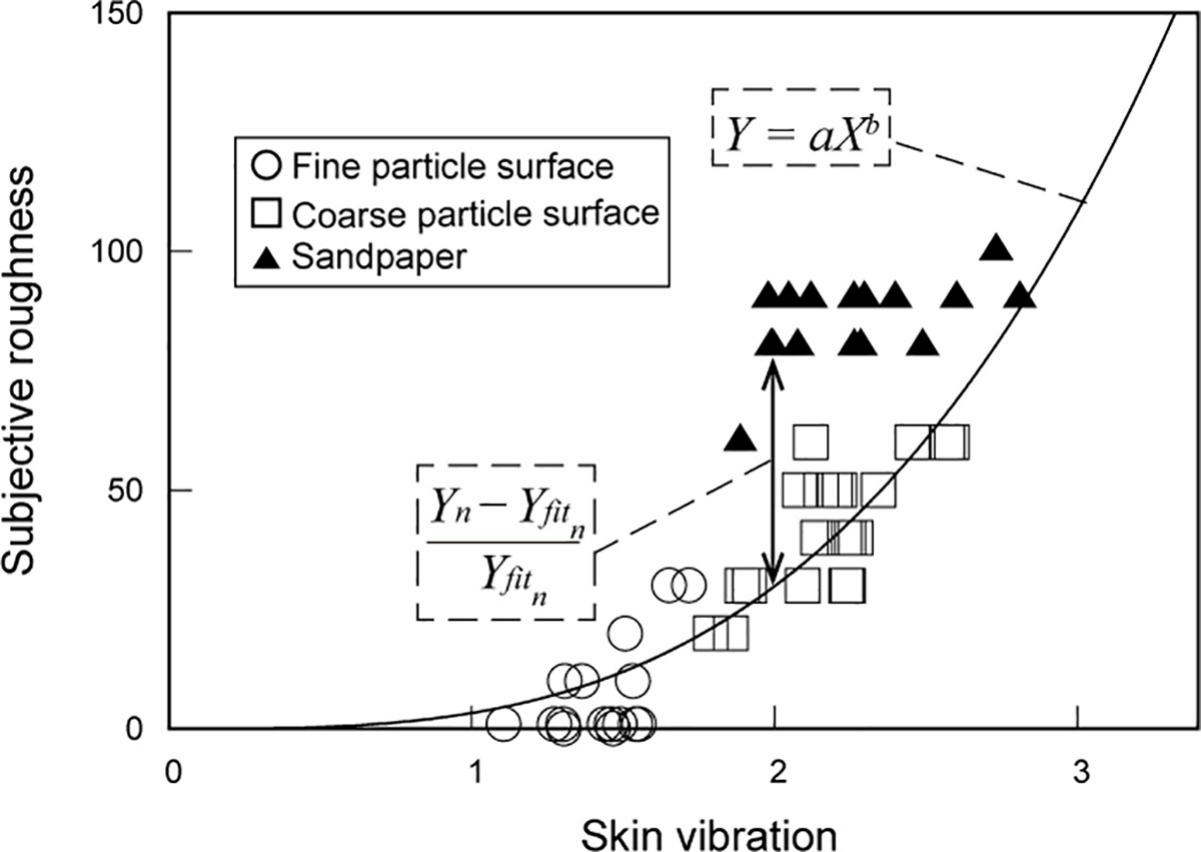
Example of the relationship between subjective roughness and skin vibration. Circles, squares, and triangles represent the data belonging to the fine glass particle surfaces, coarse glass particle surfaces, and sandpapers, respectively. The solid line shows a curve fitted to the roughness ratings of the glass particle surfaces. The vertical line with arrows shows how one data point belonging to a sandpaper rating yields the Gap, which is the difference between the subjective rating and the estimate based on the skin vibration.

Fig 3 shows a typical example for skin vibration. As mentioned before, temporal information is important for the perception of fine texture [4, 15, 19, 20, 21, 28]. Sandpapers used in the current paper were categorized as fine textures on the basis of their grain size, but other properties such as their edges and friction are quite different in comparison with the glass particle surfaces. Therefore, in the first analysis, the subjective roughness ratings of only the glass particle surfaces were fitted to particle size and skin vibration. To investigate the relationship between subjective roughness and temporal information or spatial information, the exponent of each fitted curve, which is *b* in Eq (3), was used.

Subsequently, we determined how consistent the subjective roughness ratings for the sandpapers fitted on the curve for the particle surfaces. Previous works reported that fine textures cannot be discriminated through only spatial information (static perception). Thus, mainly skin vibration should contribute to the roughness perception of the sandpapers, and not grain size. We calculated the difference between the subjective ratings of sandpapers and the power function fitted to the ratings of the glass particle surfaces as a function of skin vibration. As a measure of this difference, *GAP*_*n*_ were calculated for each sandpaper by

$${GAP}_{n}=\frac{Y_{n}-{Y_{fit}}_{n}}{{Y_{fit}}_{n}},$$ (4)

where *Y*_*n*_ is the subjective roughness estimate for sandpaper *n*, and ${Y_{fit}}_{n}$ is the computed value for the skin vibration belonging to sandpaper *n*, as shown in Fig 3. Then, the mean GAP $\sum_{n=1}^{N}{GAP}_{n}/N$ was calculated from the GAPs of all sandpaper stimuli, excluding the two references (*N* is the number of sandpapers).

Generally, it is difficult to control both surface geometry and the friction coefficient separately. In the current paper, the profile of the surface was controlled by using particle size on the surface, but the friction coefficients were mostly the same across fine and coarse textures having glass particle surfaces. Furthermore, friction coefficients of sandpapers were mostly distributed far from those of particle surfaces. For this reason, we did not fit curves of subjective ratings as a function of the friction coefficient.

In addition to the relationship between the subjective roughness and each parameter, the relationship between particle size and skin vibration was investigated with the data obtained with the fine and coarse textures of the glass particle surfaces. The results showed non-linear relationships and skin vibration was fitted to particle size by using a power function in the same way as Eq (3).

## Results

### Relationship between the various parameters

**Fig 4:**
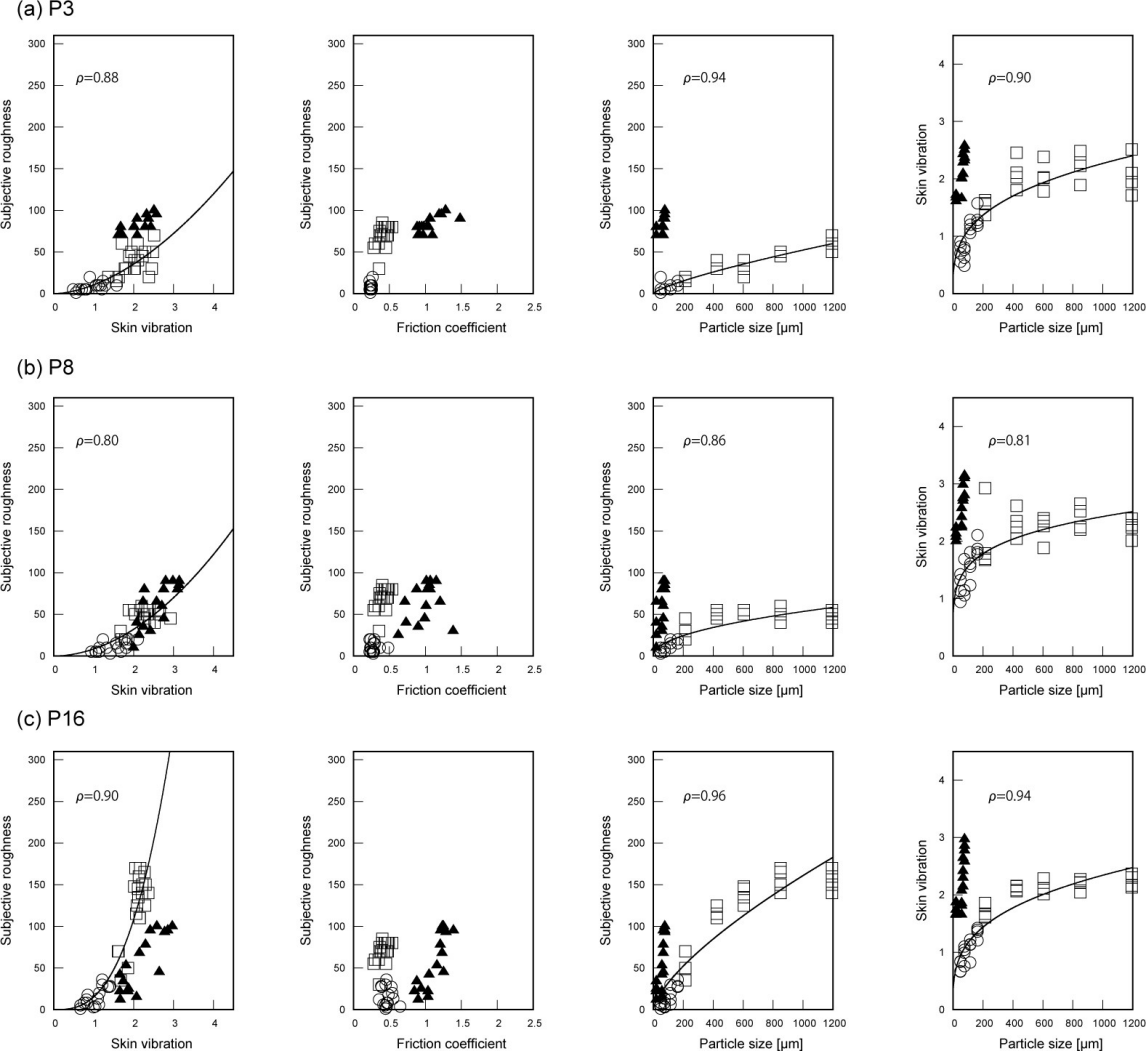
Typical distributions of subjective roughness as a function of three parameters, which are skin vibration, friction coefficient, and particle size, and relationship between skin vibration and particle size. Circles, squares, and triangles represent the data belonging to the fine glass particle surfaces, coarse glass particle surfaces, and sandpapers, respectively. The solid lines were fitted to the data of fine and coarse glass particle surfaces; Spearman’s rank correlation coefficients, which were significant for all participants with *p* values less than 0.01, are also presented.

Fig 4 shows the relationship between subjective ratings and skin vibration (1st column from the left), friction coefficient (the 2nd column), and particle size (the 3rd column) and the relationship between skin vibration and particle size (the 4th column) for three participants. In all of the graphs, circles, squares, and triangles represent the data of fine glass particle surfaces, coarse glass particle surfaces, and sandpapers, respectively. The results of all participants are shown in S2 Fig. To verify curve fitting, we calculated Spearman’s rank correlation coefficient, which is a non-parametric correlation coefficient. Correlation coefficients are presented in Fig 4 and S2 Fig. Regarding the relationships with subjective roughness, the results showed that all participants have significant relationships (roughness vs particle size and roughness vs skin vibration) with *p* values less than 0.01. The mean correlation coefficient and its standard deviation was 0.91±0.04 for roughness vs particle size and 0.83±0.07 for roughness vs skin vibration. A paired *t*-test showed that the correlation coefficient on roughness vs particle size was significantly higher than that on roughness vs skin vibration (*t*(29) = 7.2, *p* = 5.8×10^−8^). Since different exploratory movements influence skin vibration, there is some correlation between particle size and skin vibration under the exploratory movement of rubbing. The results showed that all participants have significant relationships between particle size and skin vibration with *p* values less than 0.01. The mean correlation coefficient and its standard deviation was 0.87±0.05. As follows from the exponent less than 1 for all participants, skin vibration hardly increased for large particle sizes. The exponent differed among participants: *b* = 0.28±0.07 in Eq (3).

Overall, skin vibrations well represent subjective roughness ratings on glass particle surfaces across fine and coarse textures as follows from the positive exponents and significant correlations. Roughness ratings increased with skin vibration. For different participants, the roughness ratings of sandpapers lie either above, on or below the curve fitted to the roughness ratings of particle surfaces as shown in Fig 4. As a consequence, the Gaps computed from the subjective ratings of sandpapers were different among individual participants. For most participants, the intensity of the skin vibration caused by rubbing sandpapers was larger than that of the fine glass particles with the same particle size. This might be caused by the larger friction and the sharp edges of sandpapers as compared to fine particle textures.

The exponent of the power function fitted to the subjective roughness ratings as a function of particle size is positive for all participants. However, the exponents were relatively small as compared to those of skin vibration and differed among participants as shown in Figs 4 and S2. For all participants, the subjective ratings for sandpapers lie above those belonging to the ratings of glass particles surfaces with the same particle size. Moreover, these ratings had a much wider range although the particle size hardly varied. This indicates that the roughness of sandpapers was determined by other physical parameters.

Regarding the friction coefficient, the results are quite mixed and there does not seem to be a consistent relationship with the subjective ratings. As there is a wide variation over participants, the examples in Fig 4 are not fully representative (see also S2 Fig). Perceived roughness does not seem to be systematically related to the friction coefficient; although some participants do show a relationship between the subjective ratings and friction, others do not.

### Exponents on skin vibration and particle size

**Fig 5:**
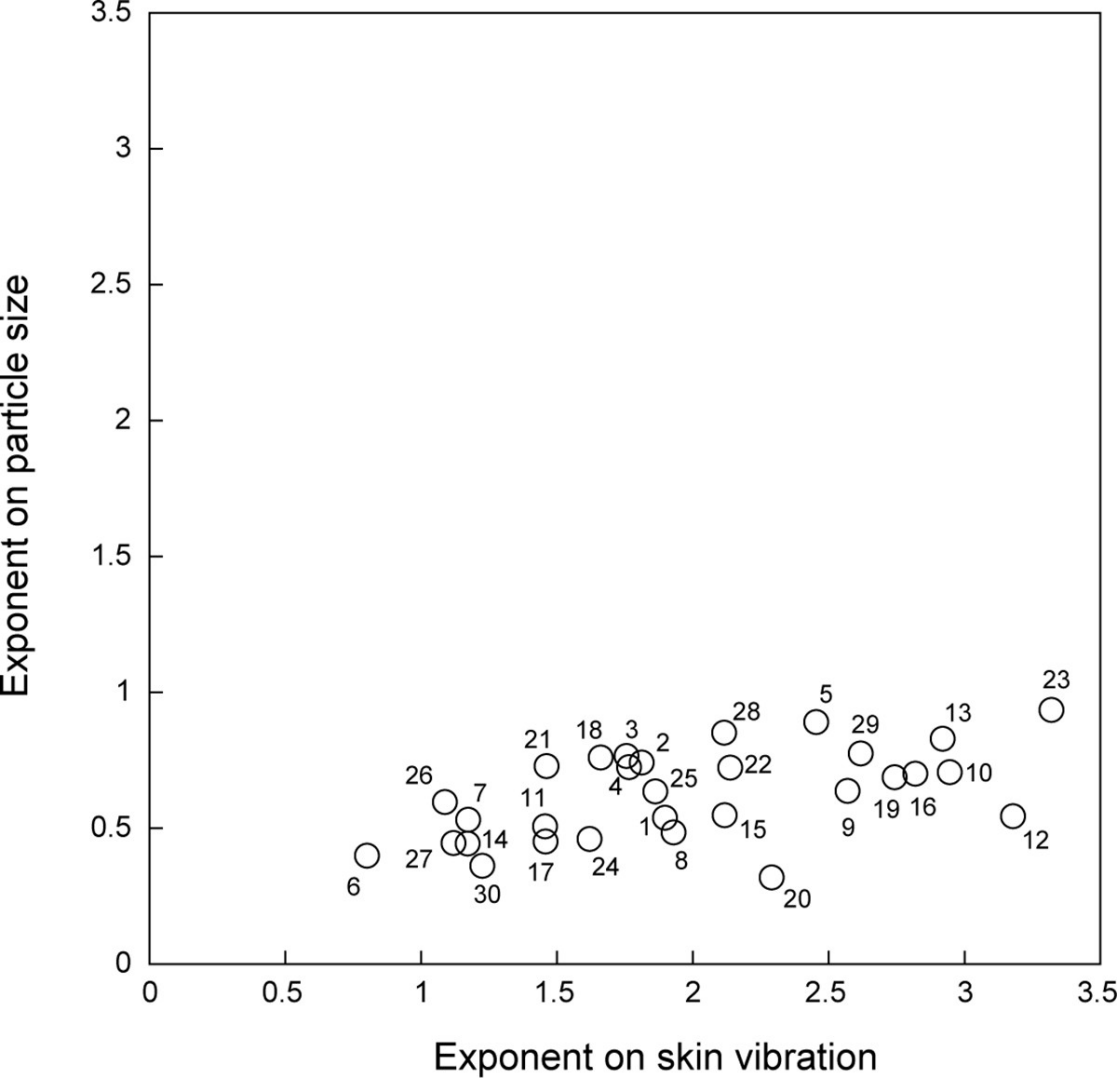
Exponents determined for particle size are plotted against those determined for skin vibration. The results of all participants are shown and the numbers near the data points indicate participant number.

Fig 5 shows the distribution of exponents determined for spatial information (particle size) vs. temporal information (skin vibration). First, all participants had positive exponents for both skin vibration and particle size with significant correlations. This result indicates all participants could evaluate the stimuli throughout the rating task. Next, it seems that exponents largely differed among participants in both spatial and temporal information. All participants had relatively large exponents for skin vibration, whereas these values differed among individuals. Regarding the particle size, some participants had large exponents around 1 for particle size and others had exponents around 0.5.

Skin vibrations basically increased across fine and coarse textures having exponents larger than 1, whereas many physical factors like particle size, friction coefficient, and edge influence the skin vibration induced and particle size also influences roughness ratings for coarse textures. Large exponents indicate that the subjective ratings greatly increase according to increase of the skin vibration and/or other physical parameters like particle size. Exponents on particle size were values less than 1 for all participants. The range of particle size is much larger for coarse textures than for fine textures. Thus, small exponents indicate that subjective ratings of coarse textures do not largely increase on large particle size.

### Gaps of sandpapers

**Fig 6:**
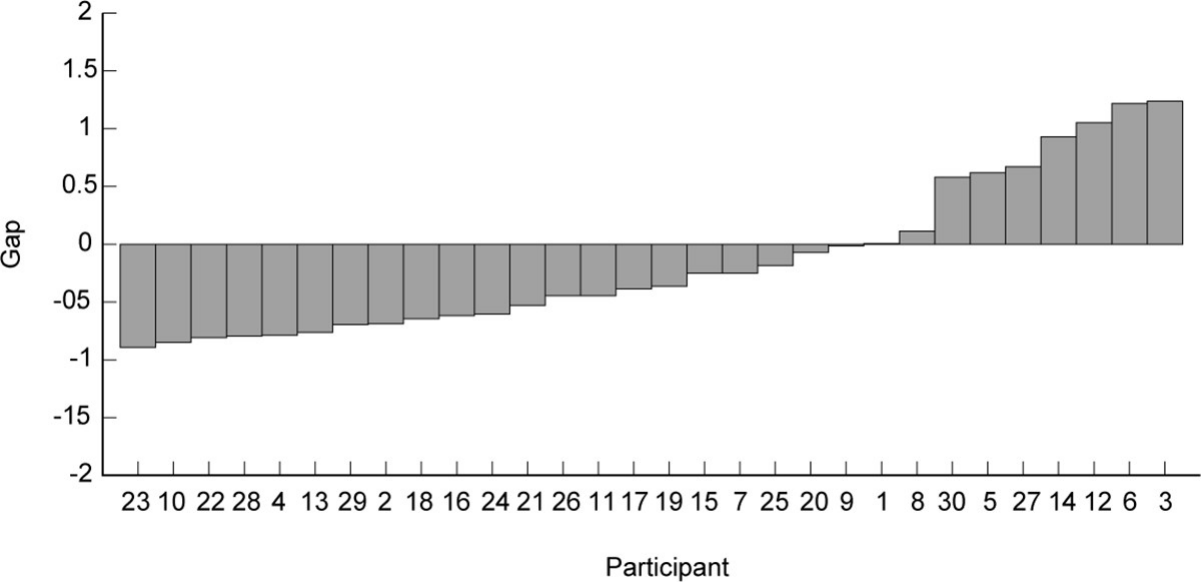
Gaps (see Eq (4)) plotted for all participants ordered by size of the Gap.

Fig 6 shows the Gaps, which indicate how much the roughness ratings of sandpapers differed from the fitted curve on the skin vibrations. The results showed almost a continuum of very small to quite substantial Gaps across negative and positive values. Large negative/positive Gaps means that particle surfaces with similar skin vibration were perceived rougher/smoother than the sandpapers. For participants with small Gaps around zero it means that the roughness of sandpapers was perceived as similar as that of glass particle surfaces with similar skin vibration. The intensity of skin vibrations on the sandpapers was larger than that of fine particle surfaces with the same particle size. Sandpapers have different properties from particle surfaces and spatial information can be involved in roughness perception for coarse textures. Indeed, the very different Gaps for individual participants clearly show that other stimulus properties, not only skin vibration, must play a role in estimating roughness for sandpapers and coarse textures.

## Discussion and conclusions

### Individual perceptual differences on fine and coarse particle surfaces and sandpapers

First, we discuss roughness ratings on particle surfaces. The correlation coefficients between subjective roughness and particle size and between subjective roughness and skin vibration were both high, with the former slightly but significantly higher than the latter. This result indicates that subjective roughness seems to closely depend on both particle size and skin vibration. However, there are clear individual differences: both the relationship between skin vibration and particle size, and the exponents of the curve fitted to roughness rating as a function of skin vibration or particle size for particle surfaces differ among individuals. Some participants had small exponents on particle size, which showed that subjective roughness slightly increases with particle size and for them particle size seems to slightly influence subjective ratings. Since humans cannot discriminate fine textures from spatial information [4], exponents on the particle size indicate how strongly the spatial information contributes to roughness ratings of coarse textures. Thus, it seems that participants having relatively small exponents on particle size might weight temporal information more strongly than spatial information (particle size) across fine and coarse textures, as can be seen in Fig 4B. On the other hand, participants having relatively large exponents on particle size, apparently use both temporal and spatial information for roughness ratings, as can be seen in Fig 4A and 4C. The inter subject differences in the relationship between skin vibration and particle size also support the possibility that participants had different weights for spatial and temporal information.

Next, for better understanding the results, we need to consider not only particle surfaces but also sandpapers since previous works reported that vibrational cues affect sandpaper roughness [20, 21] and sandpapers have different properties like relatively high friction and sharp edges. The results indicate that skin vibration well represents the roughness ratings for all glass particle surfaces. This is consistent with previous findings, which showed that temporal information correlates with both fine and coarse textures [16, 25, 26, 29]. However, the exponent of the curve fitted as a function of skin vibration was quite different among individuals. The most likely reason for this difference might be due to how different participants rated the reference stimuli. Two of the sandpaper stimuli were given to the participants as reference stimuli. As shown by the very different Gaps of individual participants (ranging from negative, via zero, to positive), the roughness of sandpaper is often determined by properties other than just skin vibration, such as possibly friction or the sharpness of the edges. Hence, participants who experience sandpapers as rougher than glass particle surfaces that caused similar skin vibrations will have positive Gaps and, as a consequence, a relatively small exponent on the skin vibration. In this case, properties of sandpapers like friction and edge might strongly influence roughness ratings, and the use of sandpapers as the reference stimuli might elicit flattening of roughness ratings of particle surfaces. There is a possibility that rougher perception of sandpapers might elicit relatively low weight on spatial information. In contrast, those participants who find sandpapers less rough than glass particle surfaces that caused similar skin vibrations will have a negative Gap and a relatively large exponent on the skin vibration. Since the intensity of skin vibrations on the sandpapers was larger than that of the fine glass particles with the same particle size, participants having large negative Gaps might weight spatial information and other properties of sandpapers more than temporal information. Exponents on the particle size for these participants seem to be relatively large as shown in Fig 4C. This tendency supports our discussion. Moreover, small Gaps indicate that mechanical properties of sandpapers hardly influenced roughness ratings. For participants with small Gaps, the skin vibration might be the important determining factor of roughness ratings for sandpapers in a way similar to fine particle surfaces. Thus, the different exponents and Gaps are a strong indication of participants using different stimulus characteristics in their judgement of roughness.

### General understanding

As the results showed, it seems that participants use different cognitive processing or weighting of the information to determine roughness ratings. How do the present results compare to previous findings? Previous works on psychophysics [4, 15, 19, 20, 21] and neuroscience [28, 29] have indicated that temporal information affects the roughness perception of fine textures. The findings by Hollins and Risner [4] indicate that temporal information does not contribute as strongly as spatial information to the discrimination performance of coarse textures. Regarding roughness estimation of coarse textures, previous studies showed that spatial information contributes to roughness ratings. It was demonstrated that subjective roughness estimation increases with groove width in classical experiments [7, 8, 9, 10]. In a recent study, it was reported that the spatial variation model, which is estimated from the spatial layout of coarse textures, accounts for perceived roughness [36]. Our study showed that temporal information relates to roughness perception for all stimuli, both fine and coarse, but that how much spatial information contributes to the roughness ratings strongly depends on the individual. Some participants weight spatial information of coarse textures much stronger than temporal information, and vice versa. However, this also strongly depended on the material, as the way the roughness of sandpapers was experienced differed among individuals. Humans are sensitive to spatial, temporal, friction, and other information. For coarse textures, humans can use both spatial and temporal information for the perception of roughness since temporal information is induced not only by fine textures but also by coarse textures [16, 21, 26, 29]. In other previous studies, influence of temporal information was demonstrated for roughness perception of coarse textures [22, 23, 24]. Moreover, the present study showed that how sandpapers were judged compared to particle surfaces strongly depends on the individual. Thus, our results are not necessarily inconsistent with previous researches.

A possible reason of large different trends among individuals might be that the reference stimuli were fine textures and/or sandpapers, and the rating task was conducted with both fine and coarse textures including the sandpapers. If the task in our experiment had been the rating of only coarse textures with coarse textures as reference stimuli, the physical factor used for the rating might have been different from the present results. In addition, the reference of sandpapers likely contributed to some of the large differences between participants who may have rated all of the particle textures smoother/rougher than the sandpaper surfaces, as discussed above. However, in daily life, we touch various textures and evaluate them. Thus, the design of our study may be relevant to demonstrate the characteristics of humans’ natural roughness estimation.

In the current experiment, the three parameters (skin vibration, friction, and particle size) inherently have some correlations under rubbing the surface and there are other possible parameters of influence in the rating of roughness, like, for example, the sharpness of edges. Therefore, from the current results, we cannot establish an estimation model integrating multiple physical factors. Additional roughness ratings by static touch or with vibrators are necessary to make an estimation model. The current paper demonstrated the presence of individual differences in roughness ratings; participants have different cognitive weights for roughness ratings across fine and coarse textures including different materials like sandpapers. Furthermore, it was considered that different materials like sandpapers might elicit different interpretations of ratings. These indicate that important cues for each person are essentially different and it will be important to consider these differences for the evaluation of roughness perception. The findings of the current paper provide useful knowledge to plan future experiments for the establishment of an estimation model of roughness ratings.

Furthermore, it is interesting to investigate possible causes of the differences in cognitive processing or weighting of the information between the participants. Biomechanical effects like the moisture and temperature of the skin (the experiment was conducted in an air-conditioned room, but there might have been small differences in the day-to-day temperature), the fingerprint structure, and the stiffness of the fingertip are related to the sensitivity of tactile sensations. Peters et al. [37] showed that fingertip size has an effect on tactile spatial sensitivity. On the basis of the current findings, it might be interesting to investigate the relationship between biomechanical effects and differences in cognitive processing or weighting of the information in future work.

## Supporting information

S1 Fig Photographs and profiles of texture surfaces. (PDF)

S2 Fig Distribution of subjective roughness as a function of three parameters, which are skin vibration, friction coefficient, and particle size, and relationship between skin vibration and particle size, for all participants, ordered by increasing Gap of the participant (see Fig 6). Circles, squares, and triangles represent the data belonging to fine particle textures, coarse particle textures, and sandpapers, respectively. The solid lines were fitted to the data of fine and courses glass particles surfaces. Spearman’s rank correlation coefficients are presented and all of them showed significant results (*p*<0.01).
(PDF)
