# Supplementary figures and images for: Individual differences in cognitive processing for roughness rating of fine and coarse textures
Source: PLoS One. 2019 Jan 30;14(1):e0211407. doi: 10.1371/journal.pone.0211407 (PMC6353187; doi:10.1371/journal.pone.0211407)

**S1 Fig. Photographs and profiles of texture surfaces**

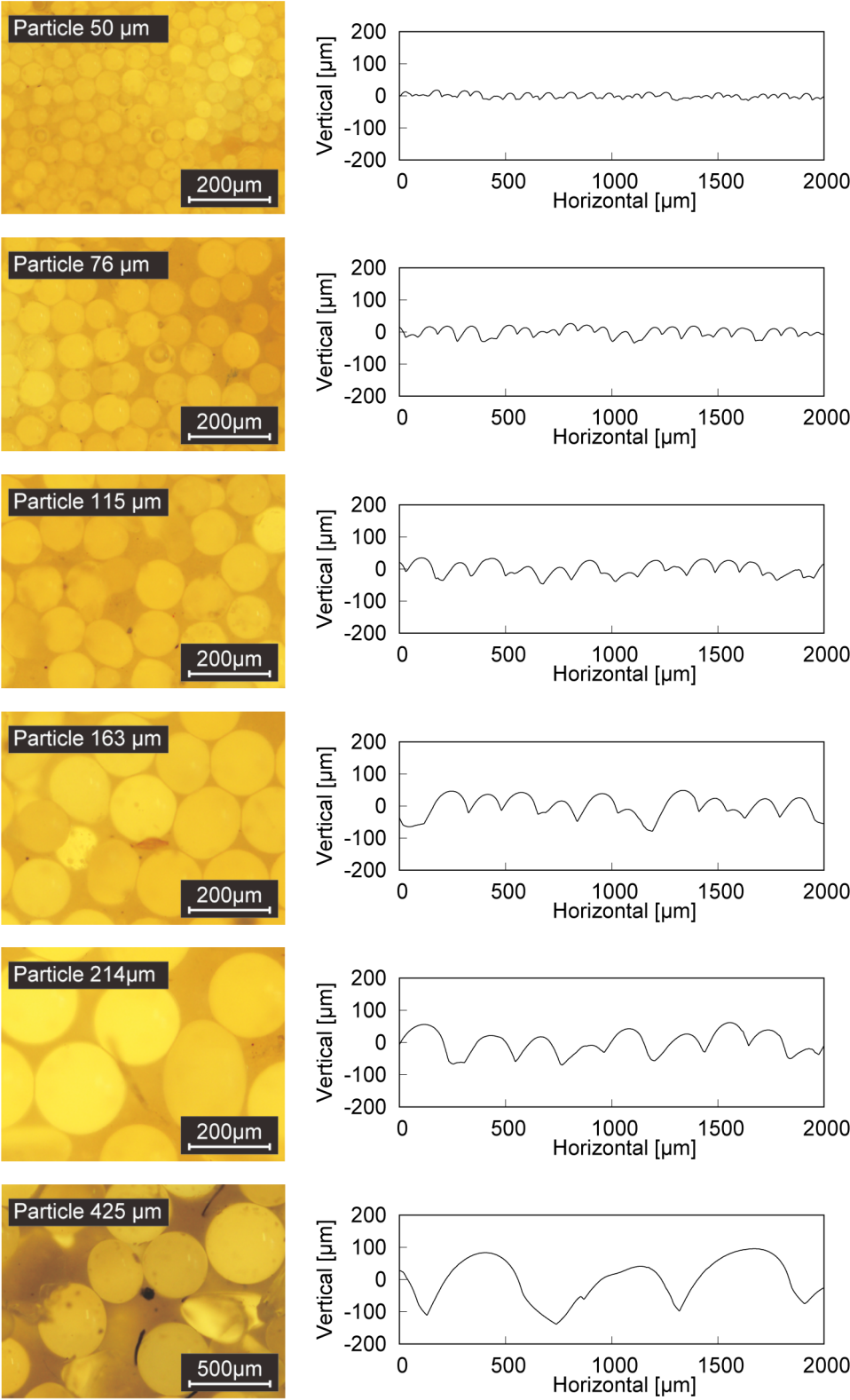

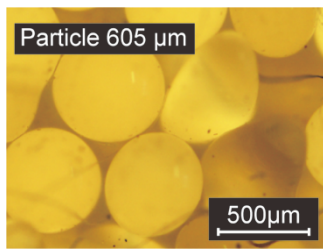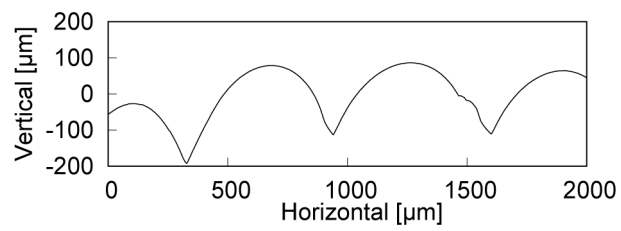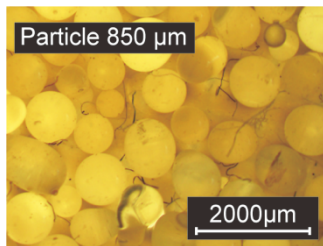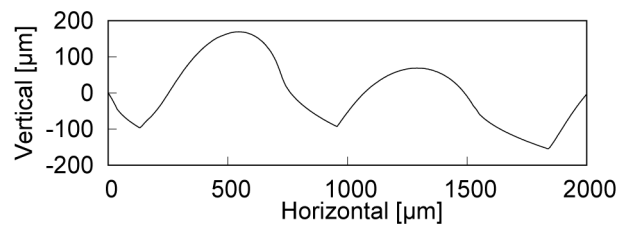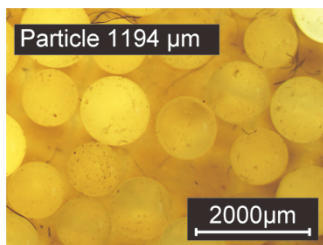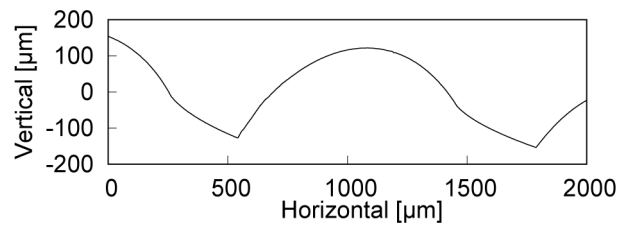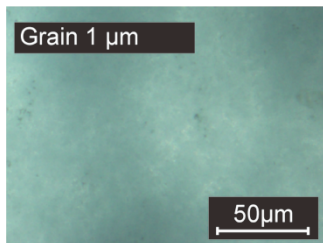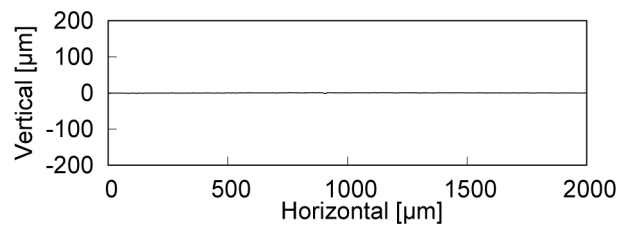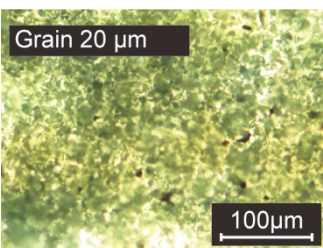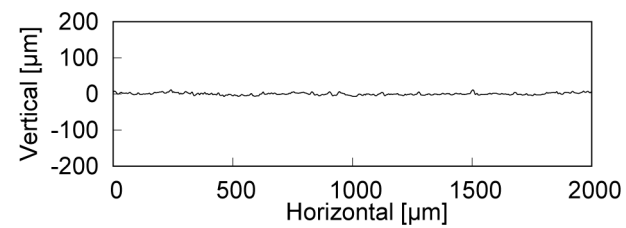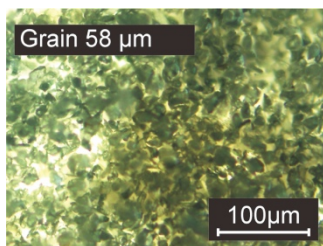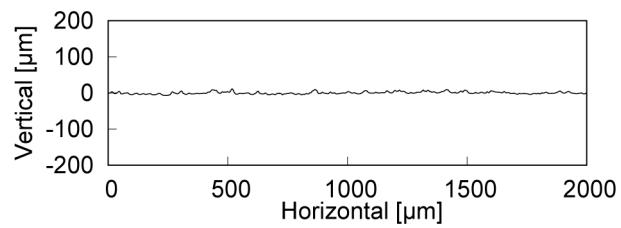

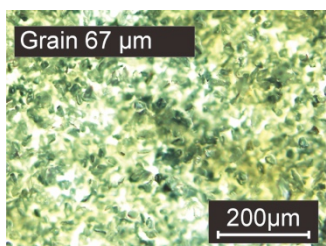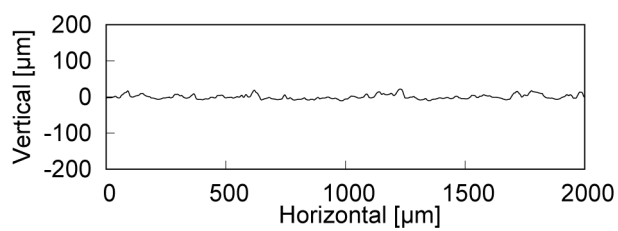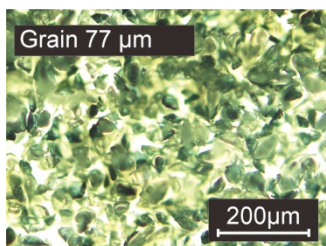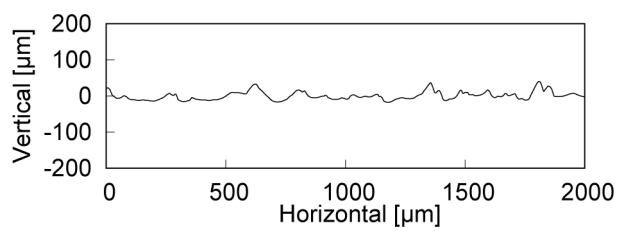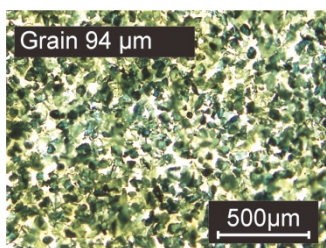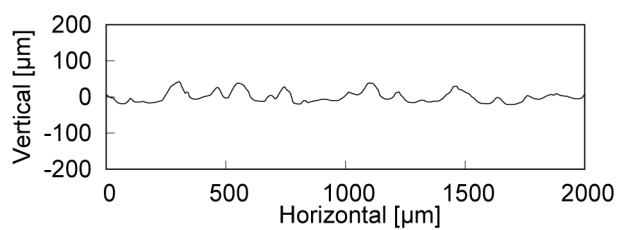

Supplement: S1 Fig — (PDF) [file pone.0211407.s001.pdf]
