# Supplementary material for: Individual differences in cognitive processing for roughness rating of fine and coarse textures
Source: PLoS One. 2019 Jan 30;14(1):e0211407. doi: 10.1371/journal.pone.0211407 (PMC6353187; doi:10.1371/journal.pone.0211407)
Supplement: S2 Fig — Circles, squares, and triangles represent the data belonging to fine particle textures, coarse particle textures, and sandpapers, respectively. The solid lines were fitted to the data of fine and courses glass particles surfaces. Spearman’s rank correlation coefficients are presented and all of them showed significant results (p<0.01). (PDF) [file pone.0211407.s002.pdf]

S2 Fig. Distribution of subjective roughness as a function of three parameters, which are skin vibration, friction coefficient, and particle size, and relationship between skin vibration and particle size, for all participants, ordered by increasing Gap of the participant (see Fig. 6). Circles, squares, and triangles represent the data belonging to fine particle textures, coarse particle textures, and sandpapers, respectively. The solid lines were fitted to the data of fine and courses glass particles surfaces. Spearman's rank correlation coefficients are presented and all of them showed significant results (p<0.01).

P23

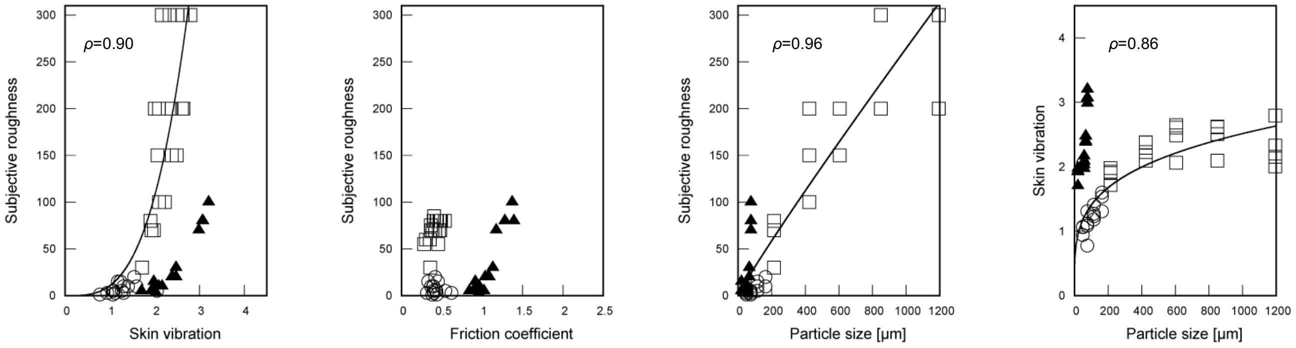

P10

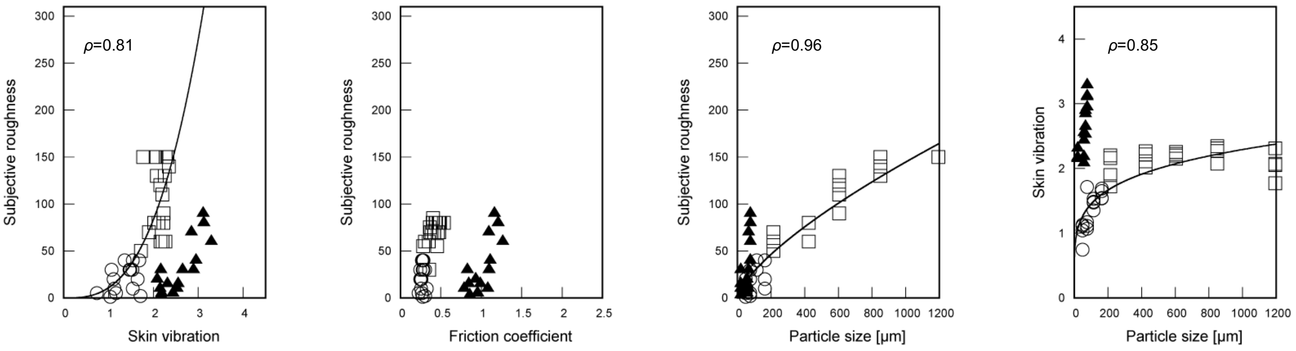

P22

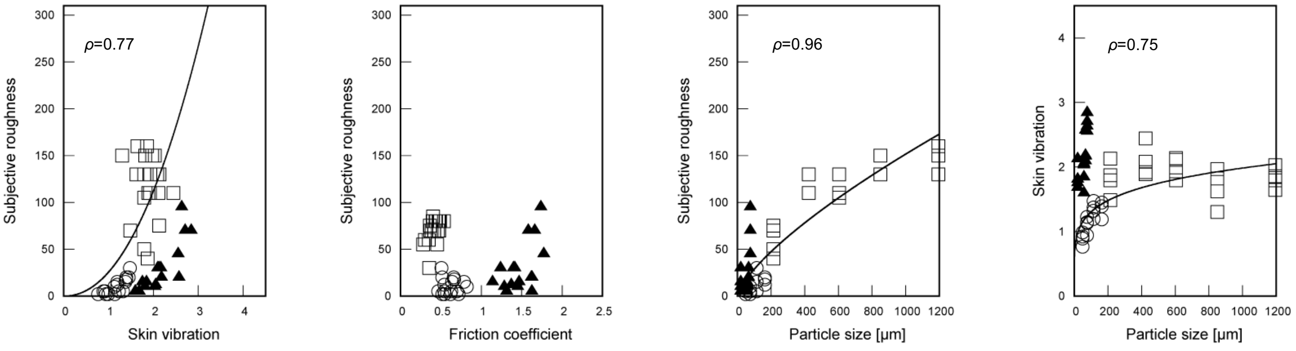

P28

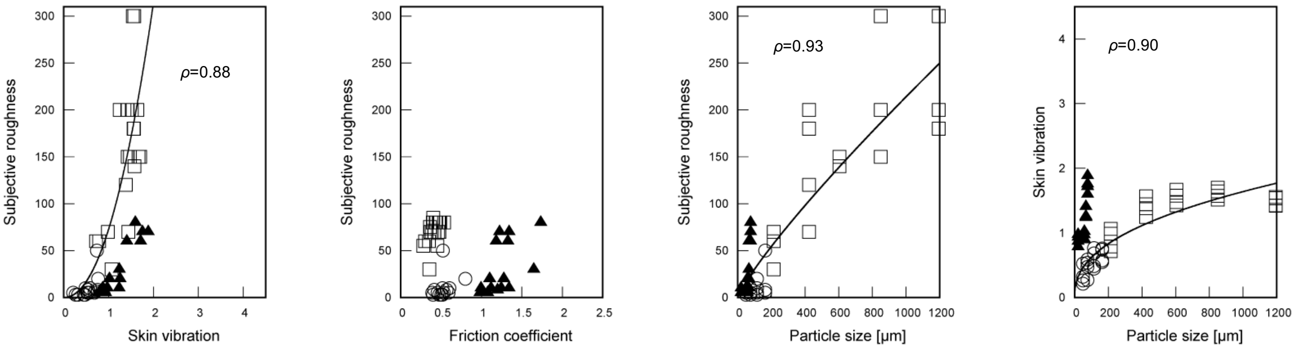

P4

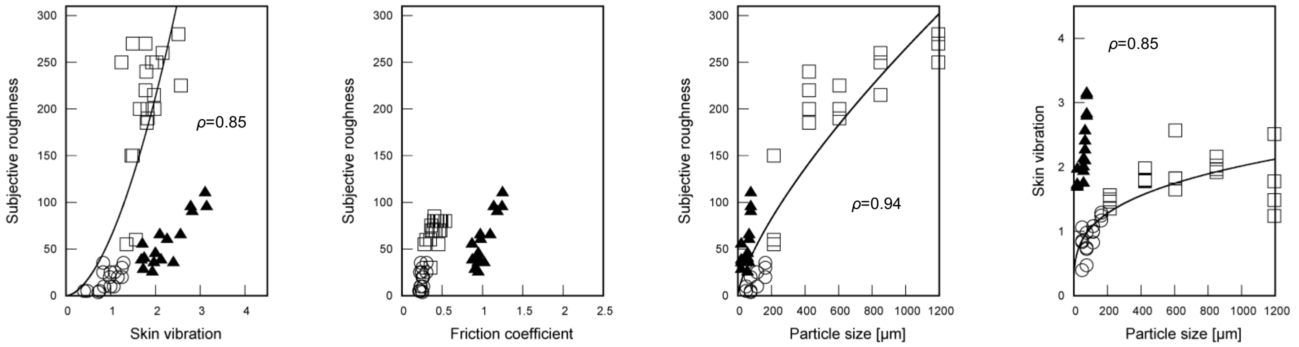

P13: note that some data point lie outside the plot range and that there were ratings up to 600.

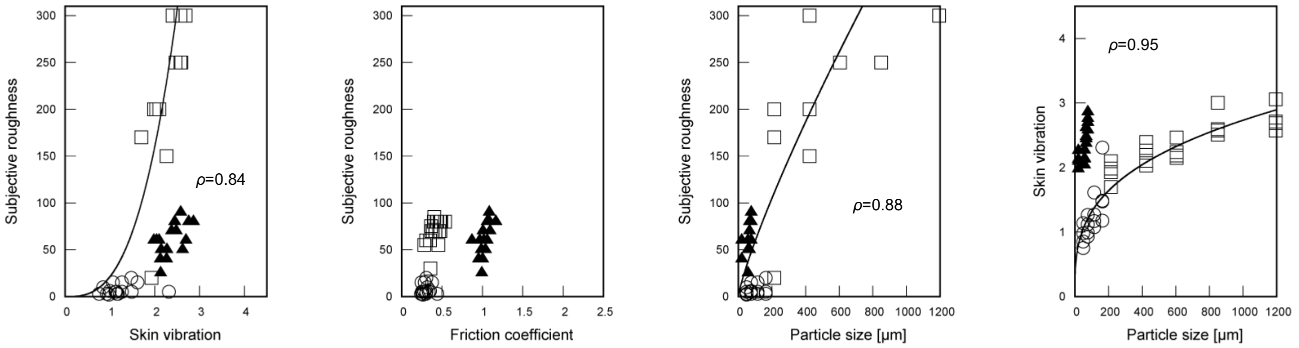

P29

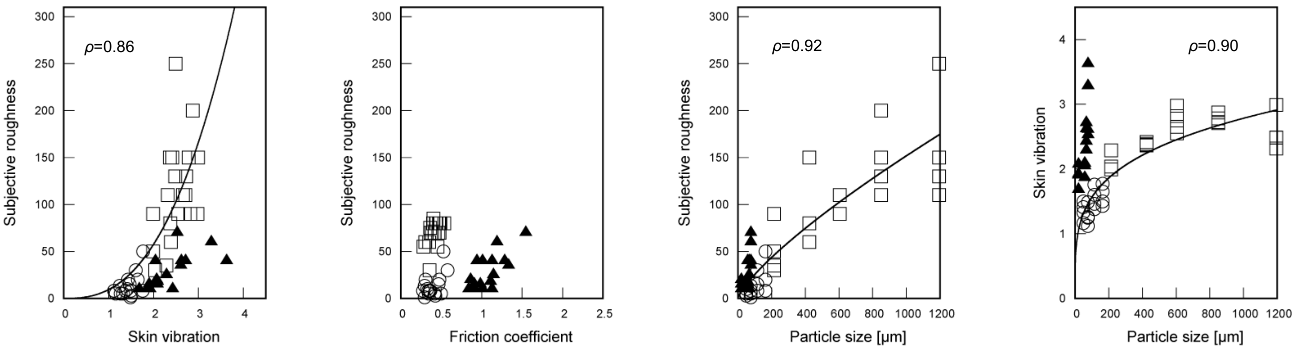

P2

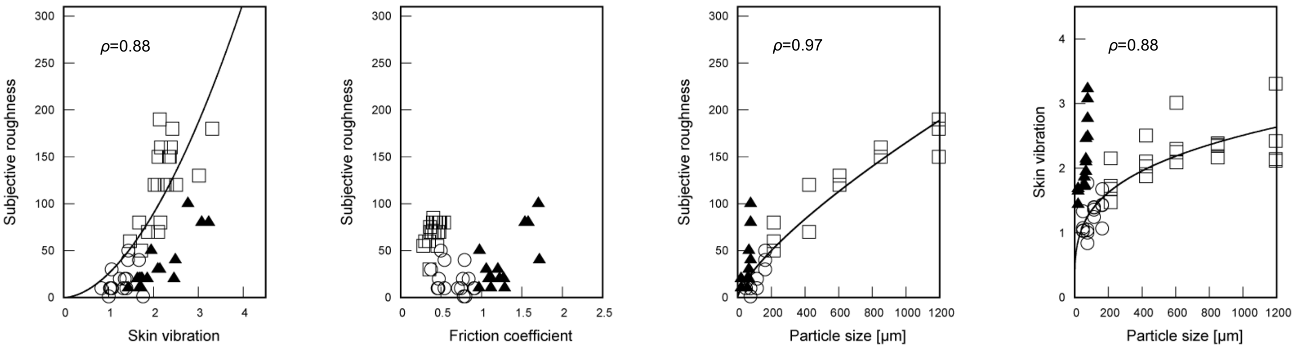

P18

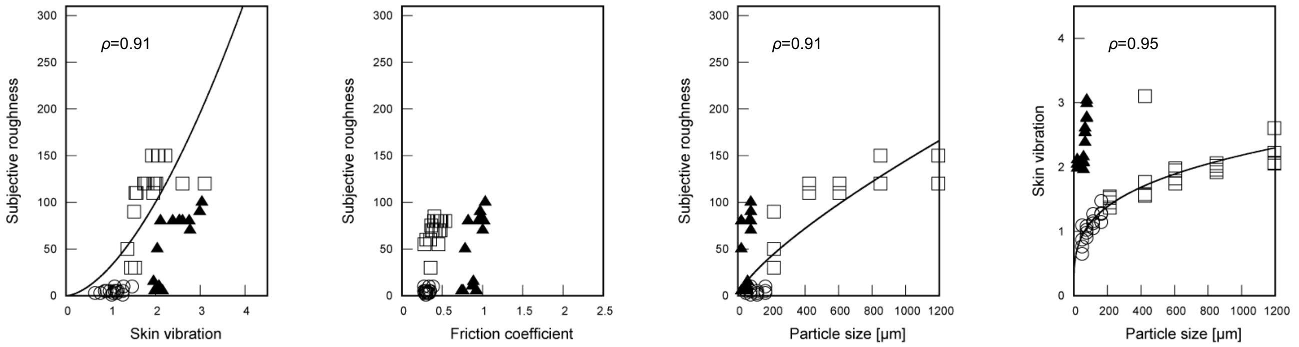

P16

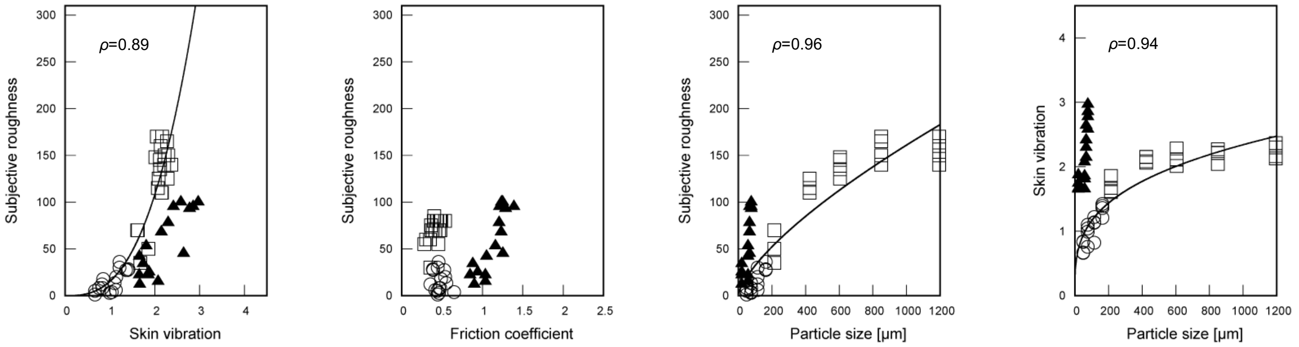

P24

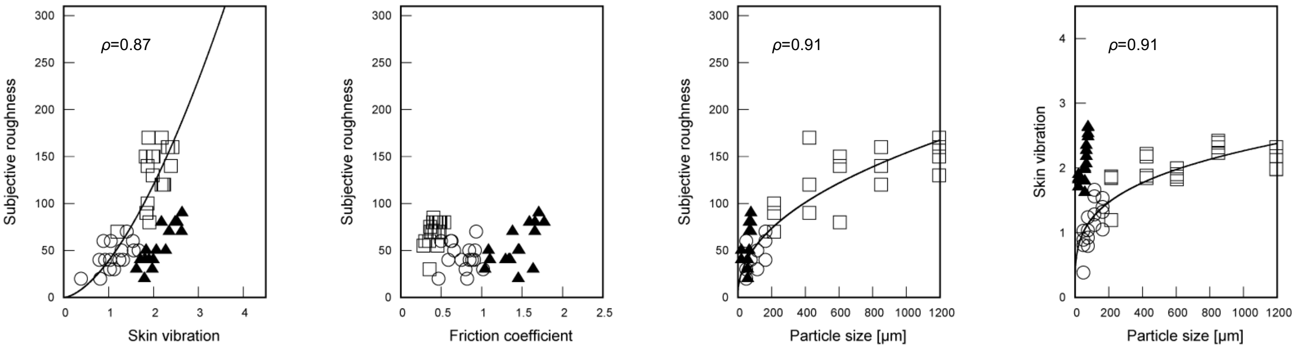

P21

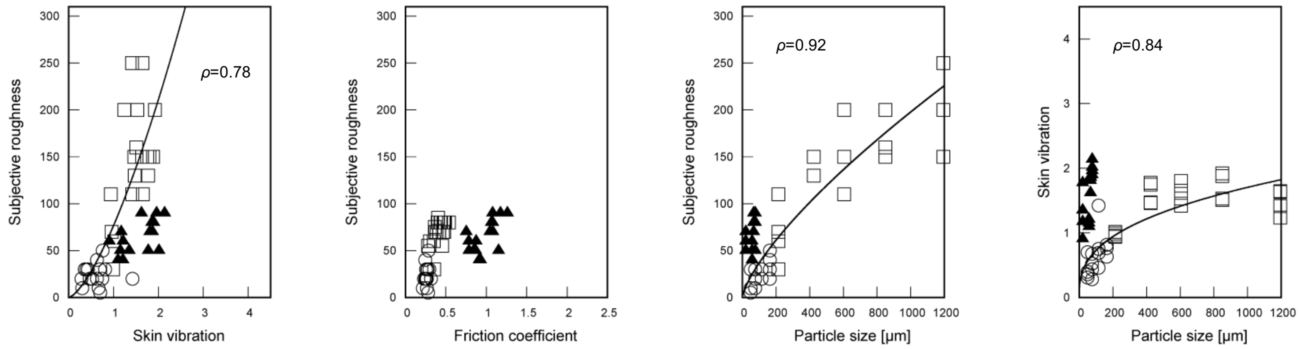

P26

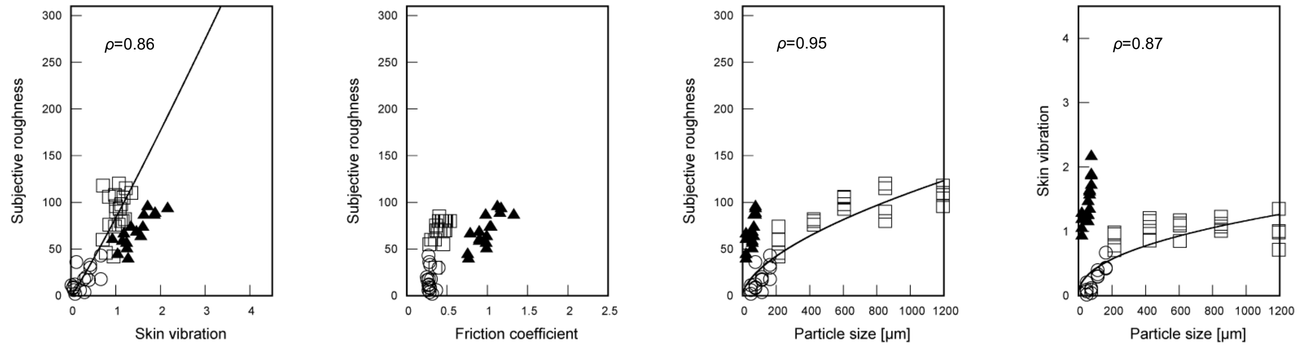

P11

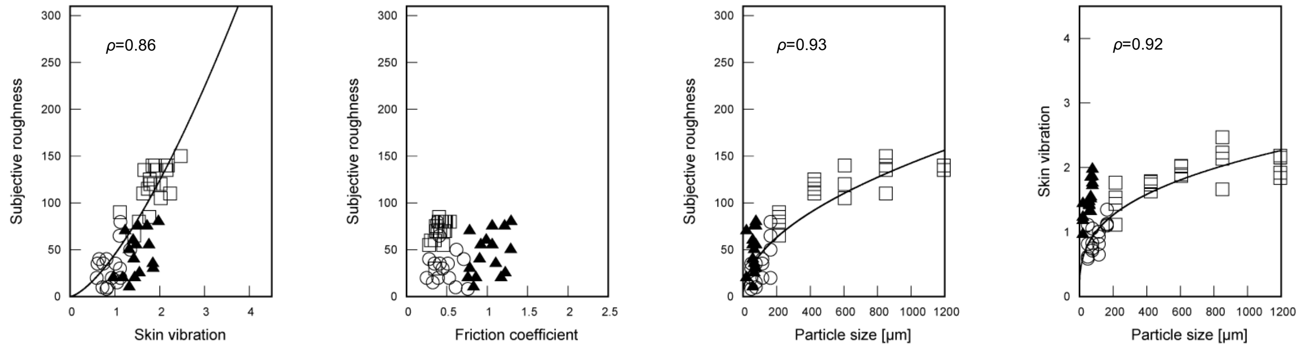

P17

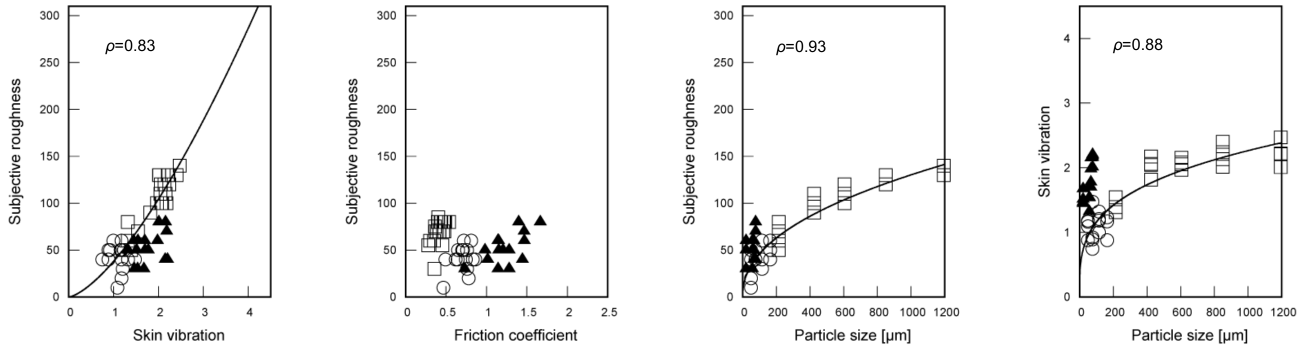

P19

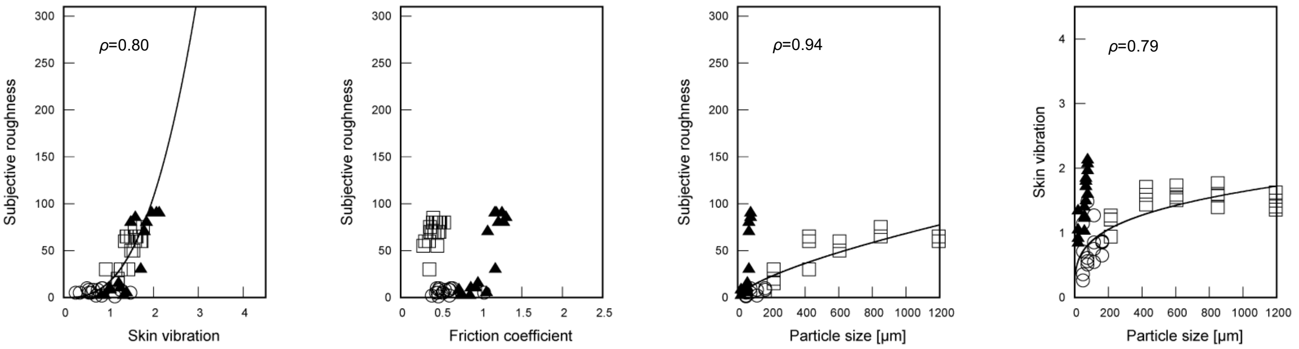

P15

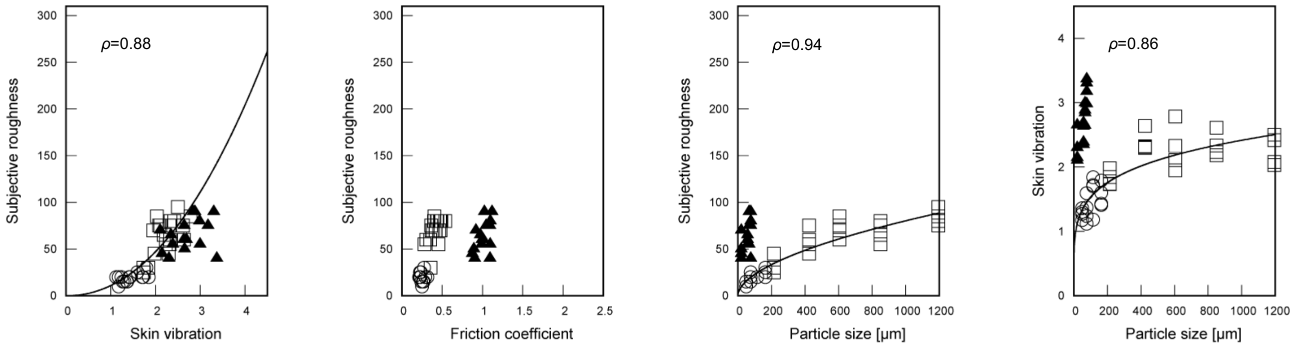

P7

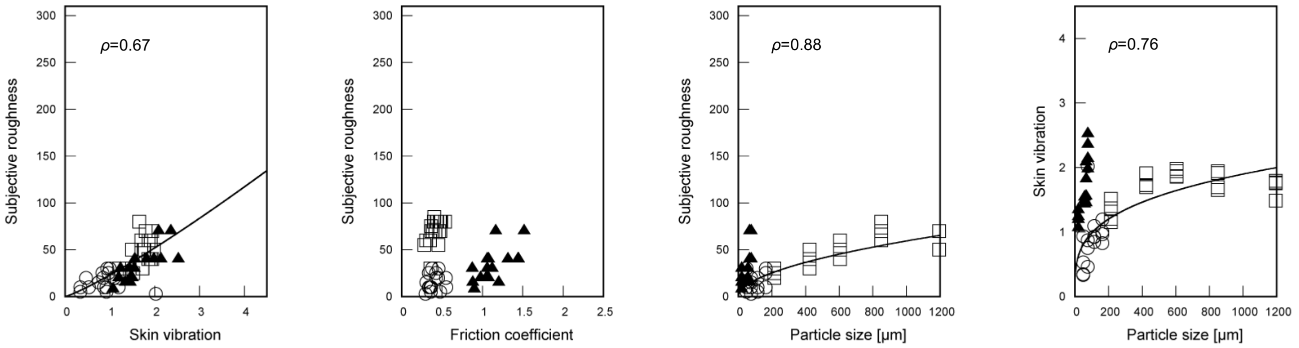

P25

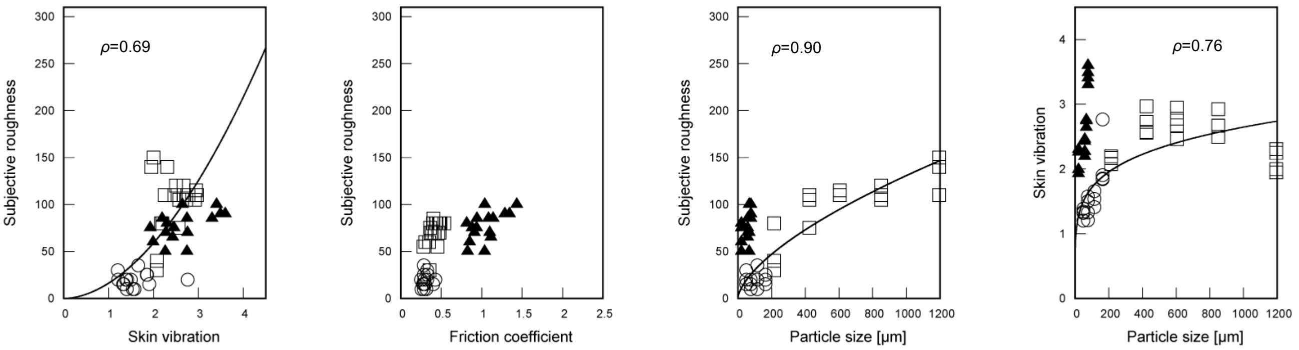

P20

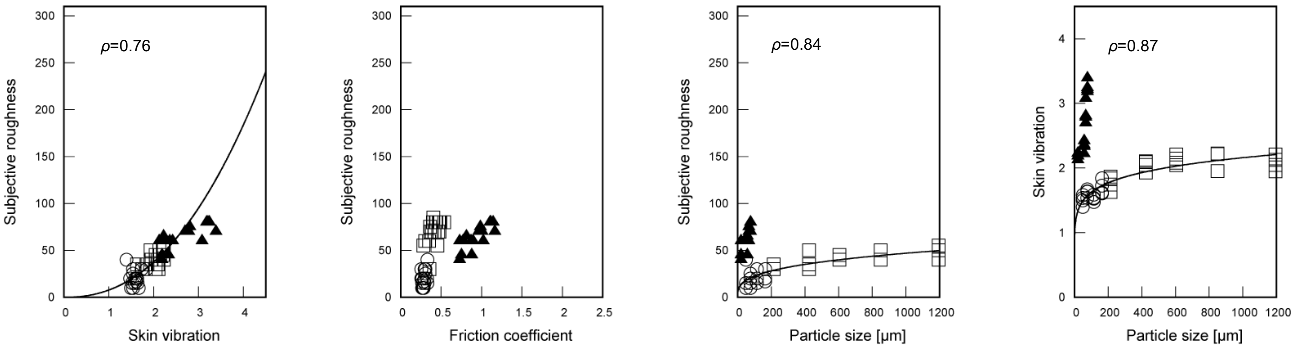
ρ=0.76
ρ=0.84
ρ=0.87
Subjective roughness
Skin vibration
Friction coefficient
Particle size [μm]

P9

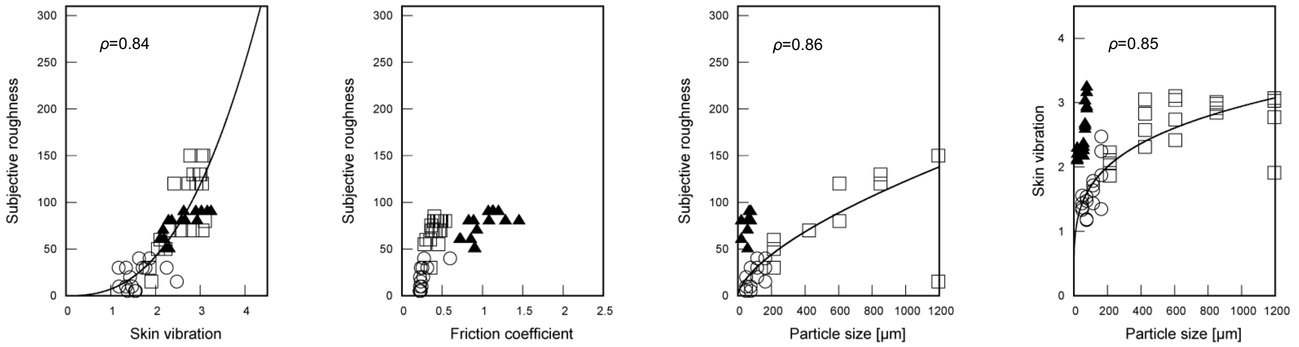
ρ=0.84
ρ=0.86
ρ=0.85
Subjective roughness
Skin vibration
Friction coefficient
Particle size [μm]

P1

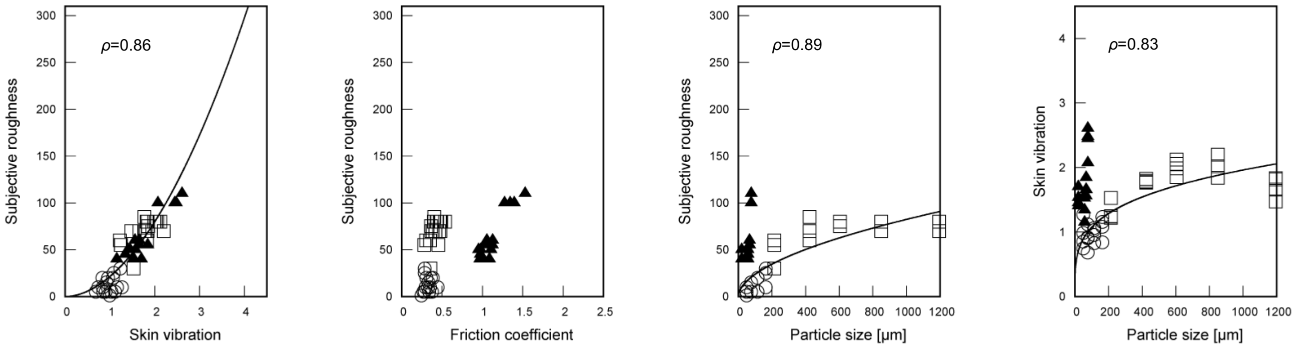
ρ=0.86
ρ=0.89
ρ=0.83
Subjective roughness
Skin vibration
Friction coefficient
Particle size [μm]

P8

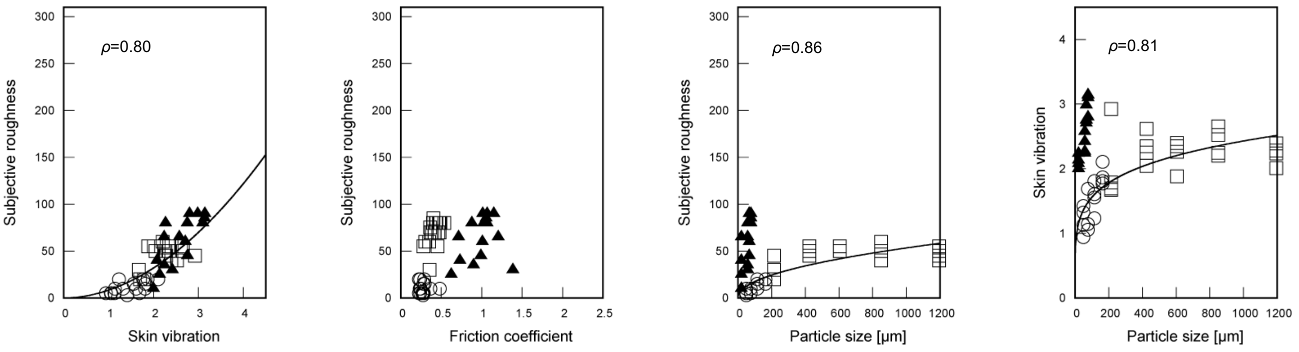
ρ=0.80
ρ=0.86
ρ=0.81
Subjective roughness
Skin vibration
Friction coefficient
Particle size [μm]

P30

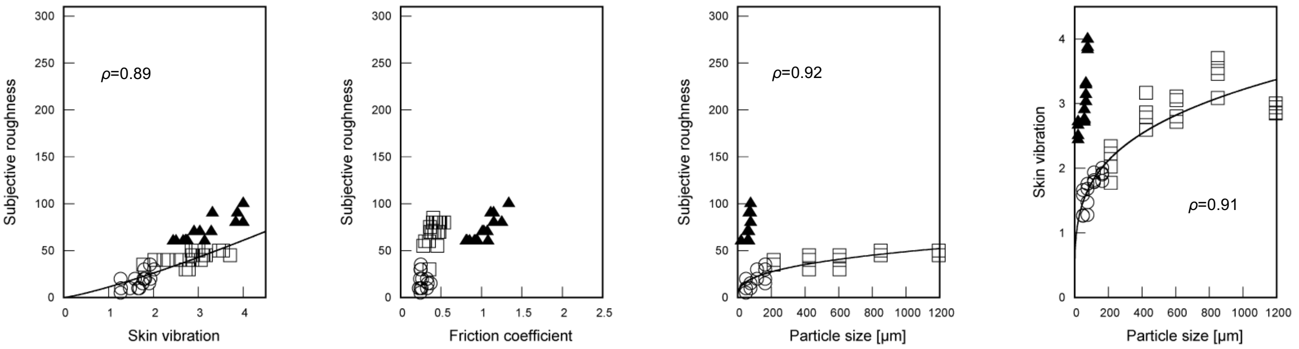

P5

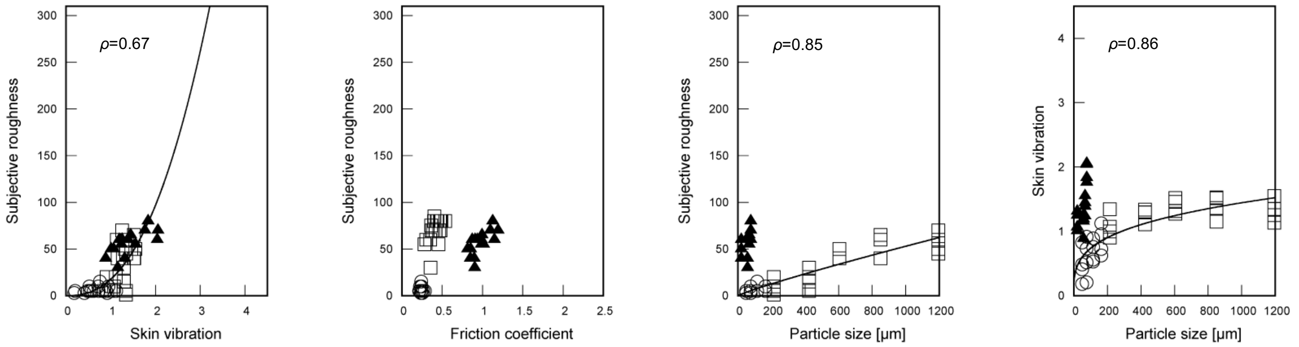

P27

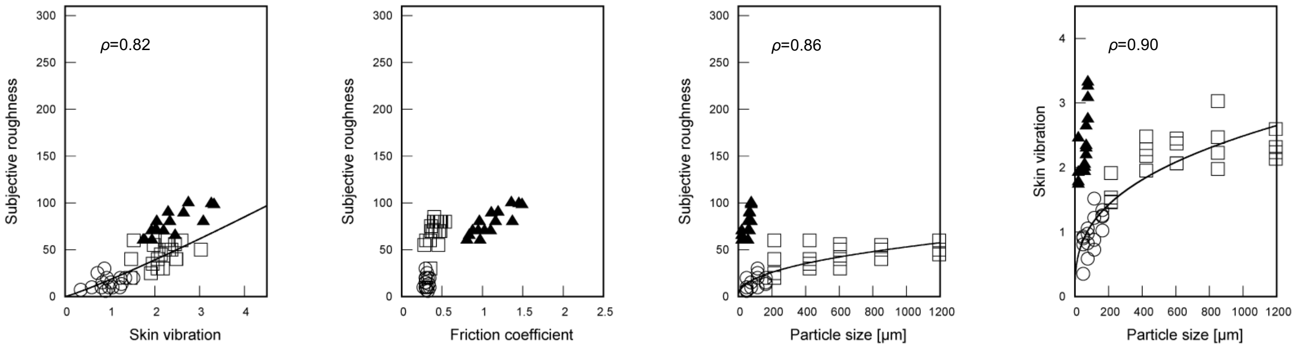

P14

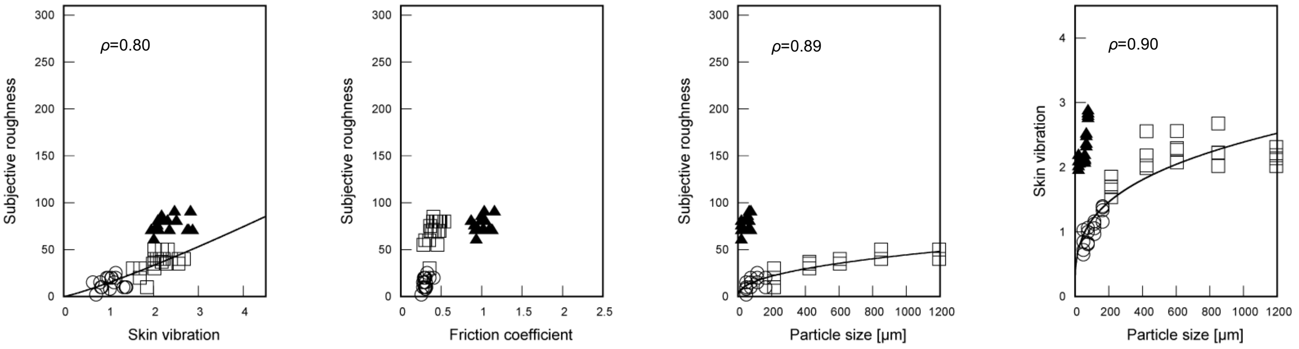

P12

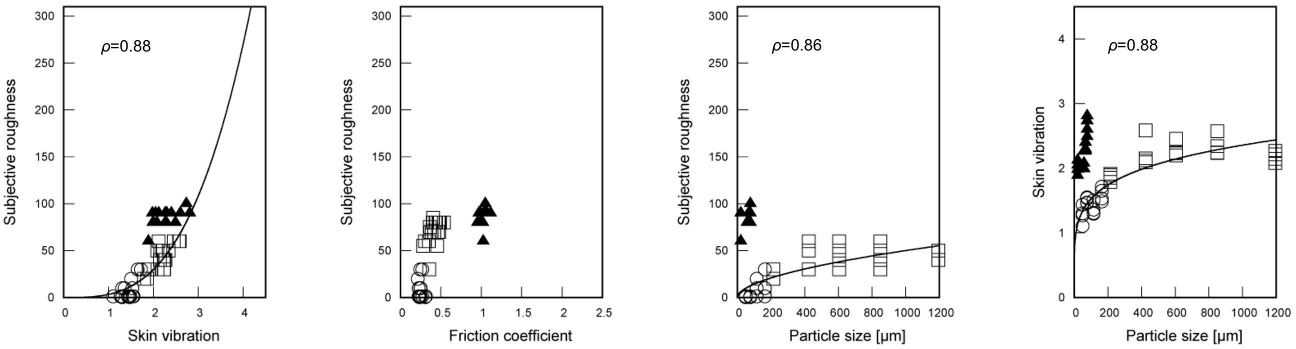

ρ=0.88
Subjective roughness
Skin vibration
Subjective roughness
Friction coefficient
ρ=0.86
Subjective roughness
Particle size [μm]
ρ=0.88
Skin vibration
Particle size [μm]

P6

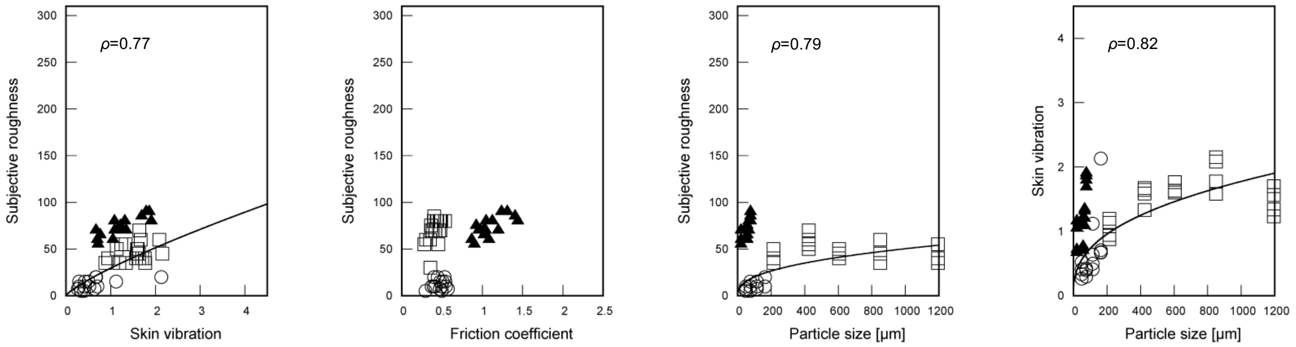

ρ=0.77
Subjective roughness
Skin vibration
Subjective roughness
Friction coefficient
ρ=0.79
Subjective roughness
Particle size [μm]
ρ=0.82
Skin vibration
Particle size [μm]

P3

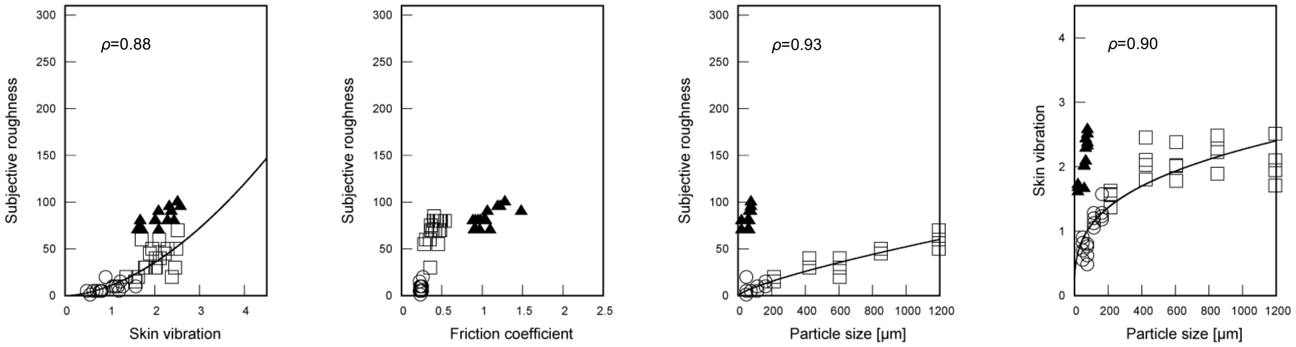

ρ=0.88
Subjective roughness
Skin vibration
Subjective roughness
Friction coefficient
ρ=0.93
Subjective roughness
Particle size [μm]
ρ=0.90
Skin vibration
Particle size [μm]
